# Impact of Environmental Innovation Strategy on Green Competitiveness: Evidence from China

**DOI:** 10.3390/ijerph19105879

**Published:** 2022-05-12

**Authors:** Na Wang, Shan Jin Zhang, Wei Wang

**Affiliations:** 1 School of Business and Management, Jilin University, Changchun 130012, China; wna18@mails.jlu.edu.cn; 2 School of Economics and Management, Changchun Science and Technology University, Changchun 130600, China; 3 Training Department, Shenyang Institute of Technology, Shenyang 113122, China; kathyweiwei1110@163.com

**Keywords:** environmental innovation strategy, green knowledge sharing, organizational green learning, green competitiveness

## Abstract

Environmental issues are a significant field in both research and practice. Manufacturing enterprises are adopting sustainable initiatives to achieve efficient resource usage, emissions reduction, energy utilization reduction, and improve waste management. Therefore, drawing on ecological modernization theory (EMT) and knowledge-based theory (KBT), this study proposes a comprehensive framework for the relationships among environmental innovation strategy (EIS), green knowledge sharing (GKS), organizational green learning (OGL), and green competitiveness (GC), through literature review; after, a survey questionnaire method was employed, and multiple-regression method was used for the analysis. The empirical results show that environmental innovation strategy has a positive effect on green competitiveness; the green knowledge sharing and organizational green learning chain mediates the relationship between environmental innovation strategy and green competitiveness. The results further reveal that green knowledge sharing and organizational green learning are crucial paths for manufacturing enterprises to enhance green competitiveness in implementing their environmental innovation strategies. This study extends previous research by emphasizing the importance of environmental innovation strategy in the context of sustainable development, and enriches existing research related to green competitiveness.

## 1. Introduction

Increasingly serious environmental problems have aroused the attention of many countries. At the just-concluded Fifth Session of the United Nations Environment Assembly (UNEA-5.2), all countries reaffirmed their commitment to integrating global environmental challenges into the overall framework of sustainable development [1]. At the 75th session of the United Nations General Assembly, Xi Jinping proposed a dual carbon goal, with China striving to reach peak CO_2_ emissions by 2030, and working to achieve carbon neutrality by 2060. As the most important developing country, China’s actions will have a great impact on the improvement in the whole world’s ecological environment, and Chinese enterprises will also play a vital role in the operation. Over the last three decades, enterprises’ environmental management has become central to good business strategy. There are very few leading businesses now that do not have environmental policies, and are not thinking hard about the impacts that they have on the environment and how they can reduce those particular impacts [2]. Enterprises need to be thinking about the impact they have on the environment and on climate change and biodiversity, and how they can make contributions to making the planet a better place [3].

Scholars have also conducted a great deal of research on environmental innovation in business to help enterprises achieve performance growth while accepting social responsibility. Hart (1995) [4] thinks that enterprises can access critical resources and achieve sustainable development by implementing proactive environmental strategies. Proactive environmental strategies encourage enterprises to accumulate resources and capabilities [5]. In industrialized nations, a growing number of companies are “going green” as they realize that they can reduce pollution and increase profits simultaneously [6]. A comparative case study of six micro-enterprises in 2012 examined the process through which micro-businesses ‘go green’, and dynamically illustrated the evolution of environmental practices over time [7]. Some studies have focused on polluting industries [8,9] and expounded on the positive impact of environmental innovation strategy on enterprise performance. The investment in green product innovation and green process innovation was helpful to businesses [10]. Some scholars identified the driving role of government, managers, and consumers on ecological innovation based on institutional theory and stakeholder theory [11], and analyzed the effect mechanism of eco-innovation on financial performance [12,13], environmental performance [14,15], and the simultaneous effect of environmental innovation on financial performance and environmental performance, based on the resource-based view [16]. Some scholars also studied the impact of environmental innovation strategy on enterprise growth [17,18]. A new study confirms that green innovation strategy helps new ventures achieve performance growth, and green knowledge sharing and business model innovation play a chain mediating role between green innovation strategy and new venture performance [19].

However, throughout the existing studies, most of the theoretical literature on environmental innovation strategy mainly focuses on the study of influencing factors or performance results, while research on enterprise green competitiveness is lacking. There are some studies on green competitiveness, but most of them focus on the macro or medium level, such as regional green competitiveness [20,21,22], provincial and municipal green competitiveness [23,24], industry competitiveness [25,26], and so on. Nonetheless, there is little research on the green competitiveness of enterprises. The study of green competitiveness for enterprises is very important. It investigates enterprises’ green marketing ability, green design ability, green production capacity, and green supply capacity [27,28].

Therefore, we study the impact of environmental innovation strategy on the green competitiveness of enterprises, and discuss the influence path between environmental innovation strategy and enterprise green competitiveness based on ecological modernization theory and knowledge-based theory. In contrast with previous studies, this paper mainly focuses on manufacturing enterprises in China, which are major environmental influencers among the world’s manufacturing powers. Their eco-friendly business strategies have a certain impact on change of the world environment, and their green innovation of products and production processes will lead to the transnational transfer of green technology, and the large-scale circulation of green products. These will be very conducive to the improvement in solutions to global environmental problems, and is worth the attention of researchers and managers.

The main contributions of this study are as follows: firstly, it expands the research field of environmental strategy. Previous studies mostly focused on the environmental strategies of small and medium-sized enterprises, or new enterprises. Since small ships are easy to turn around, the green transformation of the manufacturing industry, which contributes significantly to pollution, is ignored. In fact, due to the size of the manufacturing industry, it can have a substantial impact on the environment, which is of greater significance to the sustainable development of economic society. Therefore, this paper uses Chinese manufacturing enterprises as the research object to supplement the development of environmental strategy theory. Secondly, previous studies have verified positive correlations between environmental strategy and enterprise performance. This paper mainly discusses the impact of environmental innovation strategy on the green competitiveness of enterprises, because the improvement of green competitiveness often results in long-term development advantages for enterprises in the future. This paper also focuses on the two important paths of green knowledge sharing and organizational green learning in order to enrich the existing research conclusions on the relationship between environmental innovation strategy and green competitiveness. This study lays a theoretical and empirical foundation for Chinese manufacturing enterprises, and even manufacturing enterprises in other countries, to effectively implement environmental innovation strategies in order to enhance their green competitiveness, and puts forward valuable suggestions for promoting the green transformation of the global economy and society.

The subsequent contents of this paper are as follows: the second part reviews the existing literature, sorts out relationships between variables, and puts forward our hypotheses. The third section introduces sample analysis and data collection methods. The fourth part analyzes the data and shows the empirical research results of this paper. The findings are discussed in Section 5. Section 6 summarizes the conclusions of this paper and their implications; furthermore, we discuss limitations and directions for future research.

## 2. Theoretical Background and Hypotheses

### 2.1. Ecological Modernization Theory

The theory of ecological modernization was put forward in the 1980s, and has become a major theory of environmental sociology. This theory describes a new model: the pursuit of economically efficient, socially just, and environmentally friendly development. It is a win-win for the economy and the environment; economic growth is coordinated with environmental protection, and economic growth is decoupled from environmental pressure [29]. Eco-modernization is sometimes used interchangeably with terms such as strategic environmental management, industrial ecology, and eco-structuring; it emphasizes environmental improvements in the private sector, particularly in relation to manufacturing and related sectors [30]. The environmental problems caused by industrialization have become increasingly prominent, and it is technological progress that has led to environmental pollution and ecological damage. However, ecological modernization scholars have been positive and optimistic about technology. They believe that technology is the answer to environmental problems; the core mechanism of ecological modernization is technological and institutional innovation [31].

The practice of ecological modernization in China supports the theory of ecological modernization in some aspects, and Chinese practice suggests that there are multiple paths to ecological modernization. Mandatory government environmental laws and regulations and competitive pressures have a significant positive effect on enterprise green practices, while enterprise green practices have a significant positive effect on environmental performance [32]. A study that analyzed data from 1970 to 2014 for 137 countries found that international political integration helped reduce carbon emissions, and discussed opportunities for less developed countries to reduce their emissions [33]. In a study based on panel data of 108 cities in the Yangtze River Economic Zone from 2003–2017, technological innovation was evidenced to have a significant positive effect in promoting green development, showing a U-shaped nonlinear relationship, and this relationship varied by region [34]. As eco-modernization becomes increasingly important to all sectors, ecological modernization theory is widely used in academic and managerial fields.

### 2.2. Knowledge-Based Theory

According to knowledge-based theory, the knowledge an enterprise possesses and its ability to create and apply it is the most important source of competitive advantage [35]. It is a new type of theory that fully affirms the value of knowledge to enterprises, emphasizes the creation of an environment for employees to acquire, share, and use information, technology, business methods, wisdom, and other factors from inside and outside the organization to form personal knowledge and supports; it encourages individuals to apply and integrate knowledge into organizational products and services in order to improve the innovation capability of enterprises [36].

In 2021, China implemented a “double carbon” strategy to drive the development of green low-carbon technologies through scientific and technological innovation [37]. Green low-carbon technology innovation comes from the accumulation of green knowledge, which requires a highly participatory environment characterized by the sharing and learning of knowledge on sustainability issues and the importance of disseminating environmental information among members [38], thereby requiring green knowledge sharing and organizational green learning.

### 2.3. Conceptual Framework

#### 2.3.1. Environmental Innovation Strategy and Green Competitiveness

Environmental innovation involves the creation and exploitation of products, production processes, service, management, or business methods that are innovative, in order to reduce environmental risks, pollution, and other negative impacts of resource use compared to relevant alternatives [39]. Environmental innovation strategy is a category of manufacturing practices that includes source reduction, pollution prevention, and the embracing of environmental management systems [40]. The concept of environmental innovation strategy is very similar to green innovation strategy, sustainable innovation strategy, and ecological innovation strategy. The differences in their research content are minimal, and the terms can be used interchangeably to a large extent [41]. The environmental innovation strategy encourages the efficient use of raw materials, thus reducing the cost of raw materials and waste disposal. It actively promotes companies to develop green products and services and conduct environmentally-friendly operations. It also encourages improvement in processes and technologies to increase efficiency while reducing environmental damage [42]. By having lower costs or offering distinct products, enterprises that prioritize resource productivity, process reform, and product innovation can gain a competitive advantage over rivals [43]. Green competitiveness is a comprehensive capability of an enterprise to balance financial and environmental benefits, and it is also a key capability to achieve competitive advantage [28]. Green product innovation and green process innovation have positive effects on green product competitiveness [44].

Ecological modernization theory, as a theory of environmental innovation, is concerned with the relationship between economic development and environmental protection, emphasizing the dual benefits of the development and application of new clean technologies to the ecological environment, and business interests at the microeconomic level [45,46]. Ecological modernization theory helps us to understand and guide management innovation and change at the strategic level of the enterprise, with an environmental orientation. Environmental innovation is a vehicle for improving competitiveness, with a strong emphasis on technological progress, and on the invention, innovation, and diffusion of new and cleaner technologies and techniques [42,46]. When an enterprise focuses on an environmental innovation strategy, it increases the resources devoted to green products or processes, and coordinates these resources to achieve its goals, which in turn may help to enhance green competitiveness. Having such strategies can reduce the pressure of government policies on enterprises, and can help them develop new market opportunities. As a result of increased environmental attitudes, consumers are more willing to choose green products and even pay higher prices for environmentally friendly products [47]; this can motivate enterprises to apply green environmental protection concepts to their product design and packaging in order to cater to consumers’ preferences [48]. Based on the above arguments, we firstly propose the following hypothesis:

**Hypothesis** **1** **(H1).** *EIS is positively related to GC*.

#### 2.3.2. The Mediating Effect of Green Knowledge Sharing

Green knowledge sharing refers to the process of sharing or transferring green marketing and technical knowledge within manufacturers and among their supply chain members [19,49]. Some scholars believe that green knowledge sharing includes green supplier sharing and green customer sharing [50]; others argue that it includes sharing between members within an organization in addition to sharing between members inside and outside the organization [51]. Although scholars do not agree on the scope of the concept, there is a consensus that the transfer and sharing of green knowledge among members are essential.

In the knowledge-based view, knowledge resources are considered to be the most unique resource in the organization, and are the foundation of enterprises’ competitive advantage [52]. In essence, however, knowledge resides in the employees who create, identify, archive, access, and apply it to perform tasks. Therefore, the movement of knowledge across individual and organizational boundaries ultimately depends on the knowledge-sharing behavior of employees [51]. Effective implementation of environmental innovation strategies requires the flow of green knowledge in order to help to achieve their strategic goals, improve processes, and enhance the quality of products and services. Enterprises with an environmental innovation strategy proactively seek to acquire green knowledge by sharing and exchanging green technologies and information from outside the organization. Sharing green knowledge with other members of the supply chain network is a reciprocal practice [53]. Enterprises can integrate their own skills and knowledge with the complementing capabilities of other network members by sharing and exchanging green information and resources. As co-creators, suppliers may provide expertise or participate actively in the innovation process [54]. As an intellectual asset and intellectual capital [55], the sharing of green knowledge may bring synergistic effects to enterprises and thereby help to develop green competitiveness. Thus, we further hypothesize as follows:

**Hypothesis** **2** **(H2).** *GKS mediates the relationship between EIS and GC*.

#### 2.3.3. The Mediating Effect of Organizational Green Learning

Organizational learning is the process of generating new knowledge via external knowledge acquisition and internal knowledge sharing. Organizational green learning is different from general organizational learning in that it focuses more on the learning and application of environmental protection knowledge based on green concepts [41,56]. A similar concept to green learning is environmental knowledge learning, which is an important capability that helps enterprises to continuously improve their environmental decision support systems, and promote environmental innovation behavior [57]. The purpose of green organizational learning is to transfer environmental experience and environmental knowledge. There are many ways to transfer environmental knowledge at the organizational level, such as accelerated training and retaining retired employees as mentors, all of which are very effective. As competitors hold core environmental knowledge and technology, some enterprises, in line with the business philosophy of win-win cooperation, seek to cooperate with competitors through various channels and ways to learn from them, such as the establishment of enterprise alliance, joint organization of forums, cooperative research and development, and so on, to jointly develop the enterprise green ecosystem [58]. The long-term development of modern enterprises inevitably requires employees to actively engage in organizational green learning.

The knowledge-based theory has articulated that knowledge learning is the main factor to promote enterprises’ continuous innovation [59], and has a positive impact on enterprises’ performance [60,61] and competitive advantage [62,63]. Compared to traditional innovation, environment innovation requires more environmentally focused knowledge. Enterprises must acquire a variety of knowledge, including green knowledge about green technologies and information about green demands, which is needed to support environmental innovation [64]. To quickly put environment innovation into practice, enterprises should continuously encourage green learning among employees. Environmental innovation can reduce the marginal costs of environmental management, improve an enterprises’ environmental performance, and help enterprises build a green competitive advantage. Accordingly, we thirdly propose the following:

**Hypothesis** **3** **(H3).** *OGL mediates the relationship between EIS and GC*.

#### 2.3.4. The Chain Mediating Effect of Green Knowledge Sharing and Organizational Green Learning

Drawing on knowledge-based theory, knowledge should be coordinated within the organization, and shared outside the organization to manage and utilize the knowledge generated by organization members; the learning process plays a key role. Environmental knowledge sharing creates an environmentally friendly oriented organizational work atmosphere, encourages the willingness of employees to exchange environmental knowledge, promotes employees to generate new environmental knowledge and disseminate it, and enhances the cohesiveness of employees to master environmental knowledge [65]. Whether it is the sharing of environmental knowledge among employees within the organization, or the sharing of green knowledge among suppliers or customers outside the organization, it is necessary to transform this shared knowledge into the competitiveness of the enterprise through learning [64]. Green knowledge sharing and learning within and outside the organization make it easier for enterprises to develop a green culture, and the business sector’s mastery of this green culture becomes key to environmental healing [66,67]. In this sense, the implementation of environmental innovation strategy requires a large amount of environmental knowledge, which can be obtained through the sharing of members inside and outside the organization. Enterprises implement environmental innovation strategies and develop their green competitiveness by learning environmental knowledge and absorbing and sharing environmental information. Therefore, we fourthly assume the following:

**Hypothesis** **4** **(H4).** *GKS and OGL chain mediates the relationship between EIS and GC*.

**Figure 1:**
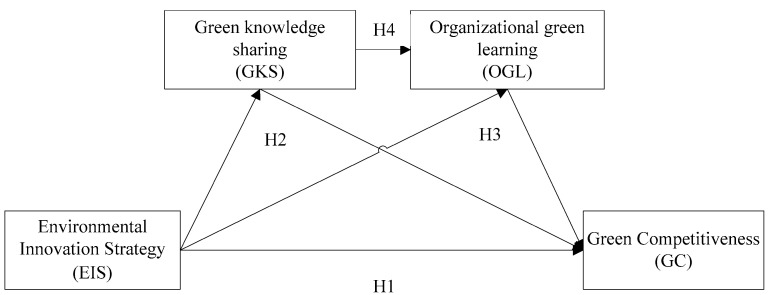
Hypothesized conceptual model.

Based on the above arguments, the conceptual model of the hypotheses proposed in this study is shown in Figure 1. The hypotheses were proposed as follows:

H1: EIS is positively related to GC.

Path: EIS → GC

H2: GKS mediates the relationship between EIS and GC

Path: EIS → GKS → GC

H3: OGL mediates the relationship between EIS and GC

Path: EIS → OGL → GC

H4: GKS and OGL chain mediates the relationship between EIS and GC

Path: EIS → GKS → OGL → GC

## 3. Methods

### 3.1. Data Source and Sample

The sample for this study was drawn from manufacturing enterprises in China. China is the world’s largest emerging economy; the effects of environmental innovation in China have a crucial contribution to overall global environmental improvement, and can also provide experience for other countries. Manufacturing is one of the industries that affects the natural and social ecology the most, leading to rapid resource consumption and serious environmental pollution. Consequently, Chinese manufacturing enterprises were chosen as the sample for this study.

We collected data for testing the research hypotheses via a survey approach, which was chosen according to the objectives and the collected data, and has the merit of relying on comprehensively processed primary sources. The use of primary data is useful for understanding what is actually happening inside Chinese manufacturing enterprises. Previous studies also provide multiple precedents for the use of such measures [66,67,68,69,70,71]. Some scholars have found that perceived firm performance data are positively related to objective data [71]. As a result, the survey method is an appropriate and acceptable method for this study.

Prior to the formal investigation, we attempted to improve the quality of the questionnaire measurement using various measures in order to ensure the scientific nature and objectivity of the sample data. Maturity scales were adopted for reference in assessing the core variables, and a 7-point Likert scale was used for evaluation. We translated and modified the foreign scales, translating them from Chinese back to English until there was no substantial difference in the meaning of the scales after the two conversions. We refined the initial questionnaire by consulting experts and conducting a pre-survey of 50 MBA students. Ultimately, our research team contacted 304 enterprise managers from July to November 2021, of which 258 responded with complete and valid questionnaires, accounting for 74.3% of the valid data.

Among these 258 respondents, senior managers accounted for 7.08%, middle managers accounted for 26.55%, and junior managers accounted for 66.37%. Listed enterprises accounted for 31.42%. For firm ages where the respondents work, 16.37% of the enterprises are established for less than 3 years, 26.11% for 4–10 years, 26.99% for 11–20 years, 12.28% for 21–30 years, and 17.70% for more than 31 years. We use the number of employees to represent the firm size; 36.28% of these enterprises have less than 100 employees, 22.57% of these have 100–500 employees, 11.50% of these have 501–1000 employees, and 29.65% of these have more than 1000 employees. High pollution manufacturing enterprises accounted for 68.58% of the firms. Table 1 displays sample characteristics.

### 3.2. Measurement of Variables

The study’s scales were designed for 4 variables, which were environmental innovation strategy, green knowledge sharing, organizational green learning, and green competitiveness. For measurement, a seven-point Likert scale was used, with “1” denoting “totally inconsistent” and “7” denoting “absolutely consistent”. Respondents were asked to analyze and select the appropriate answers according to their current business conditions. All the variable measurement scales in this study were from the mature scale, and the English-Chinese circular twice translation method [41] was used to ensure the explanatory power of the scales. Each construct was measured using items based on previously validated scales (see Table 2).

For the environmental innovation strategy, we adopted the viewpoint of Chen (2006) [10], which included 7 questions. A sample item is: “We adjusted business activities to reduce the damage to the ecological environment”.

For organizational green learning, we referred to the research results of Atuahene et al. (2007) [72], and adopted the 7-item organizational green learning scale developed by Dai Wanliang [56], among which 4 items measured exploitative organizational green learning, and 3 items measured exploratory organizational green learning. A sample item is: “One of the purposes of our search for information is to ensure that we save energy and reduce emissions and reduce environmental pollution”. 

For green knowledge sharing, we referred to the study by Bock et al. (2005) [51], Zhong and Zheng (2016) [73], Li (2017) [74], and Chang (2021) [75], to measure a total of four questions from reporting documents, industry experience, manufacturing knowledge and process design knowledge. A sample item is: “We frequently share official documents in the green collaboration process with green suppliers, such as product manuals, process flow and research methods, etc.”

For green competitiveness, we drew upon the views of Chen (2013) [27] and Wang (2019) [28], combined with the research content of this paper, and partially deleted the scale items of green competitiveness. Finally, green competitiveness included four dimensions with a total of fourteen questions. The four dimensions were green design capability, green supply capability, green production capability, and green marketing capability.

Firm size and firm age may influence green innovation [41]. Larger, longer duration firms are more likely to take risks and become innovators than younger, smaller firms [66]. Therefore, we use firm size, represented by the number of employees, and years in business, represented by firm age, as control variables for our study.

Each construct was measured and the indicators were as followed (see Table 2).

**Table 2:** Measurement scale items and indicators.

Constructs		Item Description	Loading	Cronbach’s α	CR	AVE
EIS	EIS1	We adjusted business activities to reduce the damage to the ecological environment	0.721	0.905	0.908	0.586
	EIS2	Although government regulations didn’t require it, we voluntarily took environmental remediation actions	0.814			
	EIS3	We adjusted our operations to reduce waste of resources and emissions of pollutants	0.885			
	EIS4	We adjusted our operations to achieve recycling of non-renewable raw materials, chemicals, and components	0.693			
	EIS5	We replaced traditional fuels with some new and less polluting sources of energy	0.797			
	EIS6	We adjusted our operations to reduce energy consumption	0.712			
	EIS7	We adjusted our operations to reduce the environmental impact of our products	0.718			
OGL	OGL1	One of the purposes of our search for information is to find more energy-efficient solutions to problems	0.706	0.892	0.892	0.542
	OGL2	One of the purposes of our search for information is to ensure that we save energy and reduce emissions and reduce environmental pollution	0.785			
	OGL3	We pay attention to more environmentally friendly production processes when developing new products	0.720			
	OGL4	We tend to use environmental knowledge that is relevant to exist projects	0.749			
	OGL5	One of the purposes of our search for information is to learn more about environmental protection	0.750			
	OGL6	One of the purposes of our search for information is to develop new green projects and enter new markets	0.707			
	OGL7	We collect information that is greener than technology experience in existing markets	0.733			
GKS	GKS1	We frequently share the content of green synergy work reports with green suppliers	0.754	0.795	0.813	0.527
	GKS2	We frequently share official documents in the green collaboration process with green suppliers, such as product manuals, process flow and research methods, etc.	0.715			
	GKS3	We frequently share the experience of green synergy with green suppliers	0.737			
	GKS4	We frequently share the know-how of green synergy with green suppliers in a more efficient manner	0.695			
GC	GC1	The green process design of our product life cycle is cost saving	0.727	0.944	0.944	0.549
	GC2	The design and use of our green materials is cost saving	0.803			
	GC3	We design to minimize the use of energy	0.729			
	GC4	We design products that are easy to recycle	0.720			
	GC5	We enhance the speed of upgrading green products	0.797			
	GC6	We have a choice of green suppliers	0.725			
	GC7	We have a guarantee for the supply of green materials	0.687			
	GC8	We save on distribution and storage costs	0.669			
	GC9	We carry out clean production	0.692			
	GC10	We have adopted production quality control methods and measures for green products	0.860			
	GC11	We use efficient, low-energy technologies	0.708			
	GC12	We can quickly and effectively identify the green needs of our customers	0.800			
	GC13	We have a perfect green channel	0.694			
	GC14	We implement green communication and promotion strategies	0.738			

## 4. Results

This study used Amos25.0, Spss26.0, and Process plug-in software to test the four hypotheses mentioned above.

### 4.1. Descriptive Analysis and Correlation

We used Pearson correlation coefficient analysis to examine the correlation strength and direction between variables [76]. Firm age and firm size were included in the analysis as control variables. Table 3 provides descriptive statistics and correlations for all variables. The correlation coefficients between any two core variables are significant, except for the control variables; this indicates that the hypotheses proposed in this study are reasonable and can be further tested.

### 4.2. Reliability and Validity

Reliability was assessed using Cronbach’s α and composite reliability (CR). As listed in Table 2, the Cronbach’s α values for all variables surpass the recommended value of 0.7, indicating good internal reliability. Composite reliabilities of each scale ranged from 0.794 to 0.944, which were all above the 0.70 recommended levels for acceptable reliability.

For the validity test, the measurement tools of core concepts in this study were adapted from mature research scales so that the content validity of the measurement could be guaranteed. As shown in Table 2, the results of the confirmatory factor analysis show that the factor loads of the core concept measurement questions in the study are all higher than 0.6, and the average extraction variance (AVE value) is significantly higher than 0.5, indicating that the convergence validity level of core concept measurement is also ideal. In summary, all the variables in this study have satisfactory reliability and validity.

### 4.3. Common Method Variance

In this study, questionnaires were used to collect data, which are susceptible to artificial co-variation due to social approval expectation, and to political correctness expectation under the influence of cognitive processing perspective, resulting in common method bias. Based on Podsakoff et al. (2003) [77], we adopted two methods of pre-control and post-test to reduce the influence of common method bias. For the pre-control, the questionnaire design emphasized no right or wrong answers, anonymity, and academic only to reduce common method bias, politically correctness expectation, and social desirability bias. For the post-test, Harman single factor test was first used to evaluate the factor structure of variables. Exploratory factor analysis (EFA) showed that after adding all the items of constructs into the principal component analysis, the unrotated factors solutions showed four different factors having eigenvalues above 1.0, which explained 61.52% of the total variance; the variance explained by the first common factor, 40.57%, was significantly less than 50%, and KMO was 0.935, all of which confirm that CMV was not a major problem in this study [78]. Secondly, a confirmatory factor analysis (CFA) was performed to examine the possible impact of common method bias, in which all indicators in the initial measurement validation were limited to a single factor. The fitting index of the model was poor: χ^2^/DF = 1.957, RMSEA = 0.061, IFI = 0.913, CFI = 0.912, and TLI = 0.905. Therefore, there is no serious problem of common method bias in this study.

### 4.4. Hypothesis Testing

#### 4.4.1. Direct Effect

We verified the direct effect of environmental innovation strategy on green competitiveness by using green competitiveness as the dependent variable and environmental innovation strategy as the independent variable. Model 1 in Table 4 shows a direct positive effect of environmental innovation strategy on green competitiveness (β = 0.479, *p* < 0.001); that is, Hypothesis 1 is supported.

#### 4.4.2. Mediating Effect

We used the bootstrapping method to test the chain mediation effect proposed by Hypothesis 4. We performed 5000 repetitions to determine the presence of a chain mediating effect based on whether the indirect effect includes 0 in the 95% confidence interval. The empirical results are shown in Table 5.

Table 4 reports regression analysis results of the conceptual model regarding environmental innovation strategy and green competitiveness described in Section 2. According to Model 10 in Table 4, environmental innovation strategy has a significant positive effect on green knowledge sharing (β = 0.349, *p* < 0.001). In Model 2, we can see a positive relationship between green knowledge sharing and green competitiveness (β = 0.543, *p* < 0.001). Model 4 verifies that when environmental innovation strategy and green knowledge sharing jointly affect green competitiveness, both of them still have a positive effect on green competitiveness (β1 = 0.300, β2 = 0.404, *p* < 0.001). Meanwhile, the path Ind1: EIS → GKS → GC in Table 5 shows no 0 between boot LLCI and boot ULCI ([0.034, 0.140], 95% CI), indicating that GKS mediates the relationship between EIS and GC, and the mediating effect (0.078) accounted for 17.69% of the total effect (0.441). Therefore, Hypothesis 2 is supported.

According to Model 7 in Table 4, environmental innovation strategy has a significant positive effect on organizing green learning (β = 0.401, *p* < 0.001). Model 3 shows a positive relationship between organizing green learning and green competitiveness (β = 0.622, *p* < 0.001). Model 5 verifies that environmental innovation strategy and organizing green learning jointly have a positive effect on green competitiveness (β1 = 0.247, β2 = 0.484, *p* < 0.001). Simultaneously, the path Ind2: EIS → OGL → GC in Table 5 shows no 0 between boot LLCI and boot ULCI ([0.035, 0.149], 95% CI), indicating that OGL mediates the relationship between EIS and GC, and this mediating effect (0.085) accounted for 19.27% of the total effect (0.441). Hypothesis 3 is supported.

#### 4.4.3. The Chain Mediating Effect of GKS and OGL

Model 6 shows that environmental innovation strategy, green knowledge sharing, and organizing green learning jointly have a positive effect on green competitiveness (β1 = 0.215, β2 = 0.225, β3 = 0.369, *p* < 0.001). The path Ind3: EIS → GKS → OGL → GC in Table 5 shows no 0 between boot LLCI and boot ULCI ([0.031, 0.102], 95% CI), indicating that the GKS and OGL chain mediates the relationship between EIS and GC, and that this mediating effect (0.062) accounted for 14.06% of the total effect (0.441). Thus, Hypothesis 4 is supported.

**Figure 2:**
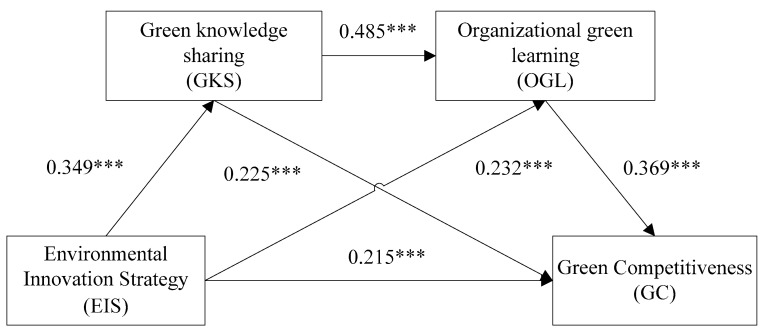
Results of path coefficients. Note: *** *p* < 0.001.

The path coefficients between the core variables are shown in Figure 2 as follows:

## 5. Discussion and Implications

Our empirical results demonstrate that environmental innovation strategies have a positive impact on green competitiveness. The findings emphasize that environmental innovation strategies are necessary for enterprises to address environmental issues and achieve beneficial environmental performance. This result also emphasizes the importance of having environmental innovation strategies for enhancing enterprises’ green competitiveness and stimulating sustainable development. Environmental innovation strategies can prepare enterprises for superior performance by enabling green modernization processes through environmentally friendly business practices. This result is consistent with Dhekra (2020) [78], who posited that eco-innovation can generate increased revenues based on differentiation strategies for improved green products. Moreover, environmentally innovative behaviors, such as recycling raw materials and reducing energy consumption, directly save costs and improve business performance. Thus, environmental innovation strategies have a crucial role in protecting the environment, improving market position, raising awareness of customer needs, and maintaining sustainable and enhanced enterprise green competitiveness.

The results of the study show that green knowledge sharing and organizational green learning mediate the relationship between environmental innovation strategies and green competitiveness, indicating that green knowledge sharing and organizational green learning are two important paths to implementing green innovation strategies to develop green competitiveness. Knowledge transfer is an activity that is promoted from outside the organization, beyond corporate boundaries, and is fostered by a culture of communication through collaboration [79]. Knowledge transfer requires the coordination of employee capabilities with enterprise infrastructure. Employees who master green knowledge share it, so that the knowledge can be learned within the organization, and other employees can also acquire green knowledge and skills; this forms a green psychological climate and a unique green organizational culture [80], guiding other members in the industrial chain to emulate this green success, thereby establishing a green image in the industry and forming green competitiveness. This is consistent with Harrison (2001) [67] and Vuong (2021) [66], who found that the business sector can reap environmental profits based on cultural values.

The chain mediating role of green knowledge sharing and organizational green learning in the relationship between environmental innovation strategies and green competitiveness was also demonstrated in the results of this study. Knowledge sharing is a process that enhances learning and also contributes to creativity [75]. In the knowledge sharing process, someone spreads the information and knowledge they know to others, which facilitates their learning and further encourages others to learn, ultimately creating a synergy [81]. Green knowledge sharing enables organization members to be enthusiastic about passing on information and knowledge about green issues to others, facilitating learning opportunities and encouraging others to learn, creating new knowledge for each other. This will help create trust and belief in the values of regeneration and in the development of natural ecosystems in the wider community [66], thereby improving the green competitiveness of enterprises [82]. A higher level of environmental literacy learning will help members of the organization to better understand the vision and behavior of environmental leaders and effectively contribute to the enterprise’s green innovation practices [60].

These findings provide some practical implications for policymakers and regulators. Government should use not only coercive instruments, such as emission standards and fines, but also incentive methods, such as financial subsidies for green products or processes, in order to effectively encourage green innovation. For enterprises implementing environmental innovation strategies, governments should consider creating an environment for them to facilitate green knowledge sharing, such as regular or occasional green innovation conferences, green knowledge-sharing platform websites, and local environmental strategy societies, in order to gain complementary green knowledge between enterprises and suppliers, customers, and even among competitors. The government should encourage enterprises to shift from an eco-deficit business culture to an eco-surplus business culture, and encourage society to educate about green practices, especially in business and economics training, to foster new green cultural values [66].

Green knowledge sharing and organizational green learning are conducive to the cooperation of supply chain members, enabling enterprises to integrate the resources of green supply chain partners, integrate the entire supply chain into industrial environmental practices, and to achieve industrial green transformation. Managers should seek talents with strong green expertise in green knowledge utilization. The efficiency of green knowledge transfer is to some extent related to the ability to learn. Managers should identify green expertise, and play their driving effect to enhance learning efficiency and achieve time advantage. Managers should encourage employees to carry out green learning and enhance their green knowledge content and green literacy by means of training or through rules and regulations, thus forming an enterprise green culture. Managers should take the initiative to promote green knowledge flow, establish connections with industrial stakeholders (customers, suppliers, competitors), and acquire new knowledge containing environmental technologies through green learning. The implementation of environmental innovation strategies requires employees to utilize learning capabilities in order to enable enterprises to absorb and integrate new environmental knowledge, thus forming green competitiveness based on green knowledge.

## 6. Conclusions

The purpose of this study is to investigate how Chinese manufacturing enterprises can improve environmental quality and reduce pollution by implementing environmental innovation strategies based on ecological modernization theory, and at the same time, to find out how to improve enterprises’ green competitiveness through environmental innovation strategies and the critical path ways. Despite extensive academic studies that analyze environmental innovation strategies or green competitiveness, the relationship between the two remains understudied, and little is known about how to influence green competitiveness through environmental innovation strategies. This study examines the impact of environmental innovation strategies on green competitiveness based on ecological modernization theory, and the mediating effects of green knowledge sharing and organizational green learning on the impact of environmental innovation strategy and green competitiveness from the perspective of knowledge-based theory. From the results, we found three aspects: firstly, environmental innovation strategies positively affect green competitiveness. Previous studies have also indirectly supported this view; Padilla-Lozano (2022) [83], Zhao (2021) [84], and Stoever (2018) [85] described the positive effects of environmental innovation strategies on green competitiveness in their studies. Secondly, we identified two important pathways to green competitiveness through environmental innovation strategies. Green knowledge sharing and organizational green learning can partially mediate the relationship between environmental innovation strategies and green competitiveness. These results were also validated by Tu (2021) [63], Zhang (2018) [62], and Song (2020) [49]. Lastly, they jointly chain mediate the relationship between environmental innovation strategies and green competitiveness. Green knowledge sharing can not only enable enterprises to obtain direct, explicit knowledge and enhance the added value of green products, but also facilitates enterprises to form their own invisible green knowledge by organizing green learning, which can influence production technologies and processes, and further enhance their green competitiveness [81].

Although the research team tried to consider the rigor of the study as much as possible, there are still some limitations. Firstly, this study used cross-sectional data. Each core variable’s changes and influencing factors cannot be judged, so the conclusions reflected by the test results are only representative of the current situation. A longitudinal study will be considered in the future to gain further insight into the critical core variables in this study.

Secondly, although manufacturing enterprises are important environmental influencers, they represent only one of them. Other industries are also sensitive to environmental strategies and need attention, such as agriculture and resource extraction; future studies will consider expanding the scope of industries studied, or even include other countries.

Thirdly, this study explored the pathways of environmental innovation strategies on green competitiveness and found mediating effects of green knowledge sharing and organizational green learning. Still, in addition, other essential forces facilitate or hinder the impact of environmental innovation strategies on green competitiveness. In the future, we will identify more influencing factors that may take advantage of environmental innovation strategy and enhance the green competitiveness of enterprise.

Finally, this study only preliminarily explores the pathways of environmental innovation strategies on green competitiveness; it does not delve into the conditions under which this mediating effect can be enhanced. We will mainly focus on this aspect in the future.

## Figures and Tables

**Table 1:** Characteristics of the sample.

Characteristics	Categories	Frequency	Percentage (%)
Position	Senior Manager	19	7.36%
	Middle Manager	71	27.52%
	Junior Managers	168	65.12%
Listed Enterprise	Yes	85	32.95%
	No	173	67.05%
Firm Age	1–3 years	43	16.67%
	4–10 years	68	26.36%
	11–20 years	72	27.91%
	21–30 years	32	12.40%
	More than 31 years	43	16.67%
Number of Employees	Under 100	92	35.66%
	100–500	60	23.26%
	501–1000	31	12.02%
	More than 1000	75	29.07%
Ownership Structure	Private Firms	74	28.68%
	Collective and State-owned Firms	127	49.22%
	Foreign-funded Firms	25	9.69%
Industry type	High pollution manufacturing	32	12.40%
	Low pollution manufacturing	103	39.92%

**Table 3:** The descriptive analysis and correlation coefficients.

	M	SD	1	2	3	4	5	6
1 EIS	5.437	1.316	1					
2 GKS	5.120	1.277	0.378 **	1				
3 OGL	5.307	1.278	0.425 **	0.580 **	1			
4 GC	5.278	1.212	0.483 **	0.544 **	0.615 **	1		
5 Firm age	2.860	1.307	0.161 **	0.167 **	0.121	0.098	1	
6 Firm size	2.345	1.235	0.102	0.117	0.185 **	0.070	0.519 **	1

Note: ** *p* < 0.01.

**Table 4:** Analysis of regression.

Variables	GC						OGL			GKS
	M1	M2	M3	M4	M5	M6	M7	M8	M9	M10
EIS	0.479 ***			0.300 ***	0.247 ***	0.215 ***	0.401 ***		0.232 ***	0.349 ***
GKS		0.543 ***		0.404 ***		0.225 ***		0.571 ***	0.485 ***	
OGL			0.622 ***		0.484 ***	0.369 ***				
Firm age	0.013	0.005	0.063	−0.025	0.025	0.002	−0.071	−0.049	−0.071	0.091
Firm size	0.014	0.004	−0.078	0.001	−0.065	−0.054	0.147	0.150	0.147	0.033
R-sq	0.477						0.4352			0.155
F	45.966						45.944			15.526

Note: *** *p* < 0.001.

**Table 5:** Analysis for chain mediation effect.

Path	Effect	BootSE	BootLLCI	BootULCI	Percent
TOTAL	0.226	0.038	0.157	0.307	51.25%
Ind1:EIS → GKS → GC	0.078	0.027	0.034	0.14	17.69%
Ind2:EIS → OGL → GC	0.085	0.029	0.035	0.149	19.27%
Ind3:EIS → GKS → OGL → GC	0.062	0.018	0.031	0.102	14.06%

## Data Availability

Some or all data and models that support the findings of this study are available from the corresponding author upon reasonable request.
